# BRCA1 deficiency leads to aberrant epithelial differentiation in the thymus

**DOI:** 10.1093/immhor/vlaf069

**Published:** 2025-12-05

**Authors:** Chikako Odaka, Marieke van de Ven, Panagiota Sotiropoulou, Yuko Baba-Sato, Andrea Karambelas, Shigeo Murata, Cedric Blanpain, Jos Jonkers

**Affiliations:** Department of Safety Research on Blood and Biological Products, National Institute of Infectious Diseases, Tokyo, Japan; Preclinical Intervention Unit, Mouse Clinic for Cancer and Aging, The Netherlands Cancer Institute, Amsterdam, The Netherlands; Institut de Recherche Interdisciplinaire en Biologie Humaine et Moleculaire, Université Libre de Bruxelles, Brussels, Belgium; Department of Safety Research on Blood and Biological Products, National Institute of Infectious Diseases, Tokyo, Japan; Institut de Recherche Interdisciplinaire en Biologie Humaine et Moleculaire, Université Libre de Bruxelles, Brussels, Belgium; Laboratory of Protein Metabolism, Graduate School of Pharmaceutical Sciences, University of Tokyo, Tokyo, Japan; Institut de Recherche Interdisciplinaire en Biologie Humaine et Moleculaire, Université Libre de Bruxelles, Brussels, Belgium; Oncode Institute and Division of Molecular Pathology, The Netherlands Cancer Institute, Amsterdam, The Netherlands

**Keywords:** stromal cells, thymus, transcription factors, transgenic/knockout mice

## Abstract

BRCA1 (breast cancer 1, early onset) is originally identified as a tumor suppressor in hereditary breast and ovarian cancer. Recent studies have suggested that BRCA1 contributes to the cell fate decisions in mammary epithelium. Although BRCA1 has been shown to be expressed in the thymus, its physiologic role(s) in the thymus remain unclear. In this study, we found that BRCA1 was expressed in a subset of thymic medullary epithelial cells: epithelial cell adhesion molecule (EpCAM, CD326) positive, UEA-1 ligand positive, and Aire negative. To clarify its functional significance, we analyzed the differentiation of thymic epithelial cells in mice in which BRCA1 was specifically deleted in K14-expressing cells. Interestingly, conditional BRCA1-deficient mice displayed enhanced development of Hassall’s corpuscles. Notably, thymoproteasome catalytic subunit β5t (proteasome subunit beta 11), a marker of cortical thymic epithelial cells (cTECs), was frequently detected adjacent to Hassall’s corpuscles in BRCA1 knockout mice. In addition, medullary β5t^+^ cells appeared to differentiate into cTECs. In addition, BRCA1 deficiency led to increased generation of regulatory T cells. Thus, BRCA1 was also found to regulate epithelial differentiation in the thymus. Our observations in BRCA1-deficient mice may be relevant to understanding the immune system in human with BRCA1 germline mutations.

## Introduction

Women with germline mutations in the tumor suppressor gene breast cancer 1 (BRCA1) have a greatly increased lifetime incidence of breast and ovarian cancer.^1^^,^^2^ Expression of BRCA1 has been demonstrated in a variety of adult tissues, including breast, ovary, testis, and thymus.^3^^,^^4^ In mouse embryos, BRCA1 is highly expressed in rapidly proliferating and differentiating cells.^4^ BRCA1 has been shown to play important roles in DNA repair, activation of cell-cycle checkpoints, and maintenance of chromosome stability.^5^^,^^6^

Mammary gland tissue contains 2 major specialized epithelial cell types: luminal cells with secretory functions surrounding the inner breast duct lumen and basal/myoepithelial cells with contractile functions that interface between luminal cells and the basement membrane. It is established that mutations in BRCA1 are preferentially associated with an increased predisposition to develop a specific subtype of breast cancer, basal-like tumors.^7^^,^^8^ A number of studies have indicated that BRCA1 functions to regulate the morphogenesis and differentiation of mammary epithelial cells.^9–11^ On the other hand, pathological examination of breast tissue samples from haploinsufficient BRCA1 patients revealed the identification of an aberrant luminal progenitor population.^12^ Similarly, mouse models with conditionally deleted BRCA1 have shown that luminal cells, but not basal cells, are predisposed to basal-like breast tumors.^13^ Collectively, these in vivo experiments have provided evidence that BRCA1-associated breast cancer arises from luminal progenitors and that BRCA1 appears to influence the cell fate specification of luminal progenitor cells. However, the precise mechanism by which BRCA1 contributes to the cell fate decisions in mammary epithelium remains to be elucidated.

The thymus, as a primary lymphoid organ for the development and maturation of T lymphocytes, plays an essential role in the acquisition of MHC restriction specificity and the establishment of immune tolerance to self-antigens.^14^ Thymic epithelial cells (TECs) constitute a major component of the thymic stroma for the proliferation, differentiation, and survival of thymocytes. TECs also include 2 main types, referred to as cortical TECs (cTECs) and medullary TECs (mTECs), according to their location in the organ. Expression of keratins 8/18 (K8/18) is largely restricted to cTECs. In contrast, expression of keratins 5/14 (K5/14) is largely seen in the medulla, with a subset of K5^+^K8^+^ cells at the corticomedullary junction and scattered in the cortical capsula. The existence of bipotent thymic epithelial progenitor cells in fetal^15^ and postnatal^16^ thymic tissues has been demonstrated. They are capable of differentiating into both cTECs and mTECs.^15^^,^^16^ TEC progenitors first emerge as early as embryonic day 11 when TECs begin to express Foxn1, and are capable of generating both cTECs and mTECs to establish a functional thymic microenvironment.^17^ However, the molecular mechanism(s) by which bipotent thymic epithelial progenitor cells expand and give rise to distinct cTECs and mTECs is still unclear.

In this work, we examined the expression of BRCA1 in the thymus of adult mice and found that BRCA1 was expressed in a subset of mTECs. To clarify the role of BRCA1 in TECs, we analyzed thymus tissues of mice with K14-expressing epithelium-specific deficiency of BRCA1. The conditional BRCA1-deficient mice showed aberrant development not only of mTECs, but also of cTECs. In addition, we found an increased generation of thymic regulatory T cells in BRCA1-deficient mice. Taken together, we suggest that the mutations of BRCA1 also influence epithelial differentiation in the thymus.

## Materials and methods

### Mice

*Brca1*F/F mice^18^ were obtained from The Netherlands Cancer Institute, Amsterdam. K14Cre transgenic mice^19^ were a kind gift from Dr E. Fuchs (Howard Hughes Medical Institute, The Rockefeller University, New York). Details of K14*cre*;*Brca1*F/F mice have been reported previously.^18^^,^^20^ Wild-type C57BL/6 mice were obtained from The Jackson Laboratory (Bar Harbor, ME, USA) or Japan SLC (Hamamatsu, Japan). The mice were housed in the specific pathogen–free animal facility at our research center. Age-matched female mice were used throughout the work. The animal care and use committee of our institutes approved the animal experiments. Experiments were performed according to the rules of our institutional animal care and use committee.

### Immunofluorescence histology

Mice were sacrificed and thymus tissues were isolated and immersed in OCT compound. Frozen thymic sections were prepared, and immunofluorescence staining was performed on thymus sections as previously described.^21–23^ The following antibodies and reagents were used: rabbit anti-BRCA1 (Signalway Antibody), rat anti-CD80 (eBioscience), rat anti-Aire mAb (RF33-1; a gift of Dr M. Matsumoto), rabbit anti-K5 (Covance Research Products), mouse anti-K8 (Progen, Heidelberg, Germany), rabbit anti-K14 (Covance Research Products), rabbit anti-involucrin (Covance Research Products), rat anti-E-cadherin (clone ECCD2) (Takara Bio), rat mAb MTS24^24^^,^^25^ (a gift of Dr R. Boyd), rabbit anti-claudin-7 (Immuno-Biological Laboratories), rat anti-mouse epithelial cell adhesion molecule (EpCAM; CD326) (BioLegend), rat anti-CD205 (Serotec), rabbit anti-β5t,^26^ rat anti-Ly51 (BioLegend), rat anti-Foxp3 (eBioscience), and Alexa Fluor–labeled donkey secondary antibodies (Molecular Probes). The binding to biotinylated *Ulex europaeus* agglutinin-1 (UEA-1; Vector Laboratories) or biotinylated *Tetragonolobus purpureas* agglutinin (TPA) (Sigma-Aldrich) was followed by FITC- or PE-conjugated streptavidin (eBioscience). Confocal images were acquired using a Zeiss LSM 510 and analyzed with Zeiss LSM software. Representative images were chosen from each experiment for figure editing. Number of fluorescence positive cells for immunofluorescence staining experiments was analyzed using ImageJ software.

### Flow cytometry

Flow cytometry analysis of thymocytes was performed as described previously.^23^ In brief, thymocyte cell suspensions were prepared, and red blood cells were removed by lysis in ammonium chloride buffer. After washing, cells were stained with directly labeled antibodies against CD3 (FITC-conjugated), CD4 (eFluor450-conjugated), CD8 (V500-conjugated), CD44 (APC-Cy7–conjugated), and CD25 (PE-conjugated). All antibodies were purchased from BD Biosciences. One hundred thousand live cells were analyzed on a BD LS-Fortessa flow cytometer using FACSDiva 2 software (BD Biosciences).

## Results

### BRCA1 is expressed in a subset of mTECs

**Figure 1.**
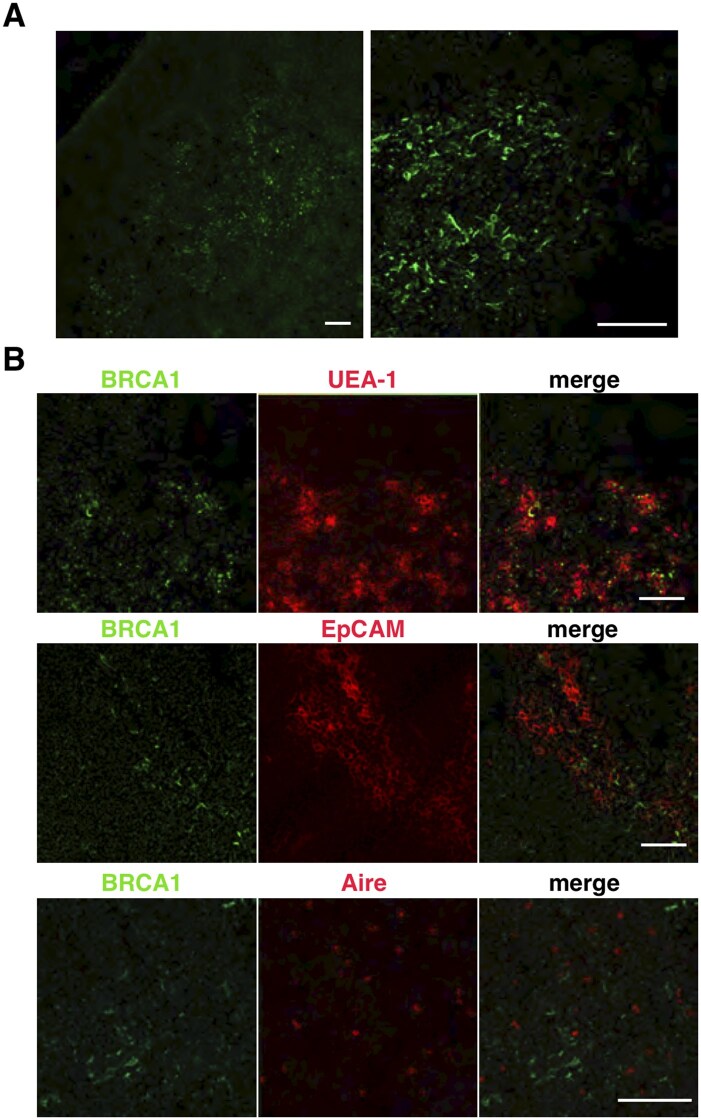
BRCA1 is expressed in a subset of mTECs. Immunofluorescence staining of thymus sections from 7-week-old wild-type C57/BL6 female mice was performed to detect BRCA1 (A, B) and the binding of UEA-1, EpCAM, or Aire (B). Note that BRCA1 is expressed in UEA-1^+^EpCAM1^+^ mTECs but not in Aire^+^ mTECs. Data are representative of independent experiments (*n* = 6). Scale bars = 100 µm.

To study the function of BRCA1 in the thymus, we first determined the localization of BRCA1 in the mouse thymus. Thymus sections from 7-week-old C57BL/6 mice were labeled with anti-BRCA1 antibody and analyzed by confocal microscopy. The sites of BRCA1 expression were located within the thymic medulla (Fig. 1A). Thymus sections were then stained with the lectin UEA-1, which specifically recognizes mature populations of mTECs. BRCA1 was present within UEA-1^+^ mTECs (Fig. 1B). The high levels of EpCAM expression delineate the medullary epithelial compartment.^27^ Most BRCA1-expressing mTECs stained positive for EpCAM (Fig. 1B). The autoimmune regulator Aire is expressed in mature mTECs, and these cells are largely postmitotic.^28^^,^^29^ Aire^+^ cells were scattered throughout the thymic medulla, but Aire expression was barely detectable in BRCA1-labeled mTECs (Fig. 1B). Thus, most BRCA1-expressing mTECs stained positive for UEA-1 ligand and EpCAM, but not for Aire.

### BRCA1 deficiency in K14^+^ cells leads to enhanced mTEC differentiation

We have previously generated mice carrying conditional *Brca1*F alleles in which *Brca1* exons 5–13 are flanked by *loxP* sites and crossed these mice with K14*cre* transgenic mice to produce mutant animals with epithelium-specific loss of BRCA1, K14*cre*;*Brca1*F/F mice.^18^ To clarify the role of BRCA1 in the organization of thymic epithelium, we used *Brca1*F/F and K14*cre*;*Brca1*F/F mice. First, we confirmed the absence of BRCA1 expression in the thymus of K14*cre*;*Brca1*F/F mice (data not shown).

**Figure 2.**
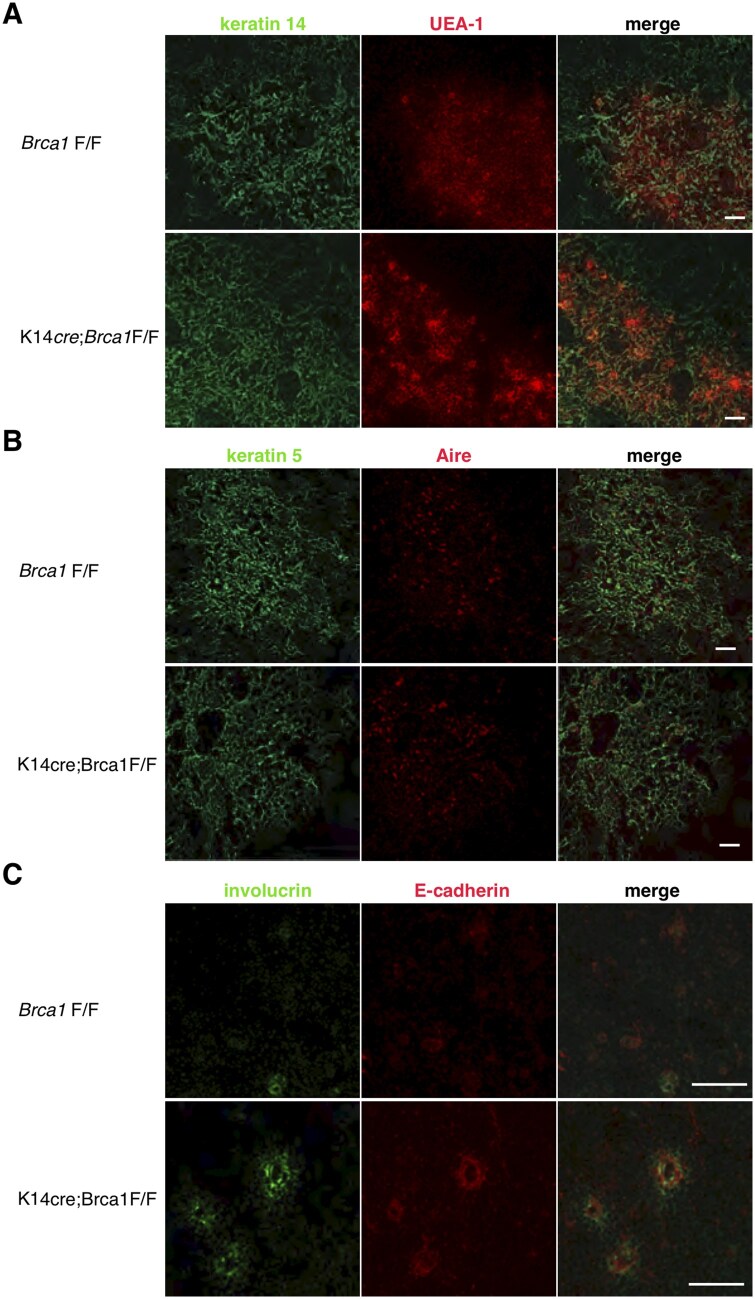
BRCA1 conditional knockout mice display enhanced mTEC differentiation. Immunofluorescence staining of thymus sections from 7-week-old *Brca1*F/F and K14*cre*;*Brca1*F/F female mice was performed to detect either keratin 14 (A), keratin 5 (B), or involucrin (C) with the binding of UEA-1 (A), Aire (B), or E-cadherin (C). In K14*cre*;*Brca1*F/F mice, the strong binding of UEA-1 is frequently detected in globular cell bodies (A). Involucrin-expressing globular structures are frequently seen within the thymic medulla of the conditional BRCA1 knockout mice (C). Data are representative of independent experiments (*n* = 8 in each group). Scale bars = 100 µm.

We stained thymus sections from both mice with reagents recognizing mTECs and analyzed the differentiation status of mTECs by confocal microscopy. K14 expression allowed the distribution of mTECs in both mice (Fig. 2A). In the conditional BRCA1 knockout mice, the strong binding of UEA-1 was frequently detected in the form of globular cell bodies. As expected, the expression pattern of K5 corresponded to that of K14 in the medullary region (Fig. 2B). Aire expression was not significantly different between the 2 mice (Fig. 2B). Hassall’s corpuscles, also known as Hassall’s bodies, are thought to be composed of terminally differentiated mTECs.^30^ Hassall’s corpuscles are not typically seen in mouse and rat.^14^^,^^22^ Intriguingly, we found larger involucrin-expressing spherical epithelial structures in the thymic medulla of K14*cre*;*Brca1*F/F mice, compared to those of *Brca1*F/F (Fig. 2C) and K14*cre* mice (data not shown). These Hassall’s corpuscles in K14*cre*;*Brca1*F/F mice varied in size, and large globular cell bodies were frequently detected. The epithelial cells composing the layers of Hassall’s corpuscles showed high expression of E-cadherin (Fig. 2C).

**Figure 3.**
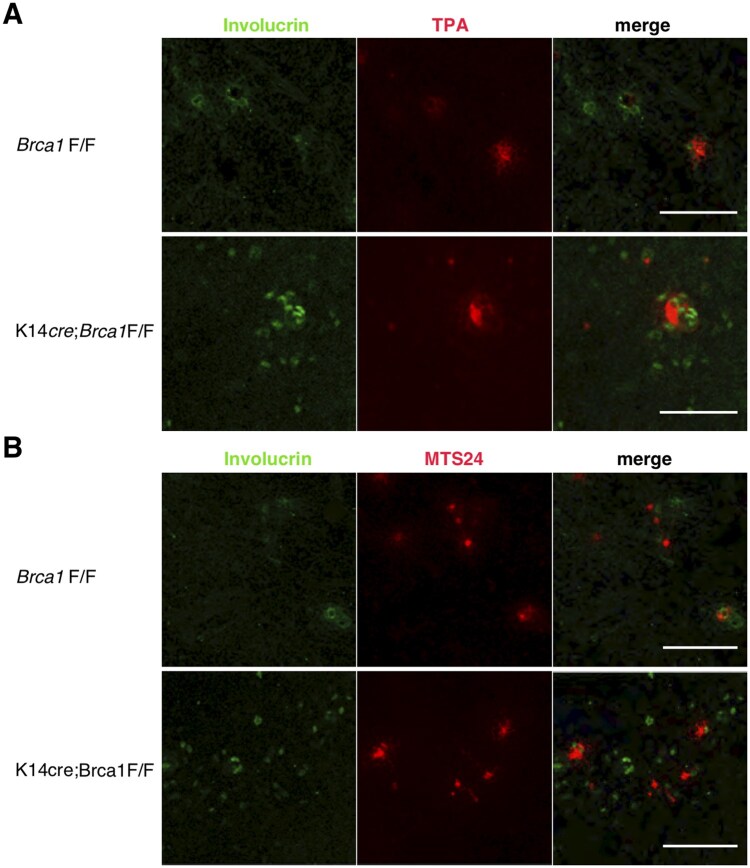
Development of Hassall’s corpuscles in BRCA1-deficient mice. Immunofluorescence staining of thymus sections from *Brca1*F/F and K14*cre*;*Brca1*F/F female mice was performed to detect involucrin (A, B), the binding of TPA (A), and MTS24 (B). Hassall’s corpuscles are frequently detected in BRCA1-deficient mice. Data are representative of independent experiments (*n* = 8 in each group). Scale bars = 100 µm.

In parallel, we assessed the development of Hassall’s corpuscles in K14*cre*;*Brca1*F/F mice using the lectin TPA and MTS24 as the relevant markers (Fig. 3). TPA particularly binds to Hassall’s corpuscles,^23^^,^^31^ and we observed many involucrin^+^ Hassall’s corpuscles in K14*cre*;*Brca1*F/F mutant mice (Fig. 3A, B). The mAb MTS24 identifies a population of cells that can differentiate into both cTECs and mTECs and is therefore considered a putative epithelial progenitor marker.^24^ During early embryonic development, MTS24 is expressed at high frequency in thymic nonhematopoietic cells,^25^ whereas most MTS24 staining is observed in Hassall’s corpuscles in the postnatal period.^22^ Involucrin-expressing cells were frequently detected adjacent to MTS24^+^ cells in BRCA1 knockout mice (Fig. 3B). Collectively, these results indicate that BRCA1 deficiency in K14^+^ TECs induces enhanced development of Hassall’s corpuscles.

### Reduction of EpCAM/claudin-7–expressing mTECs in BRCA1-deficient thymus

**Figure 4.**
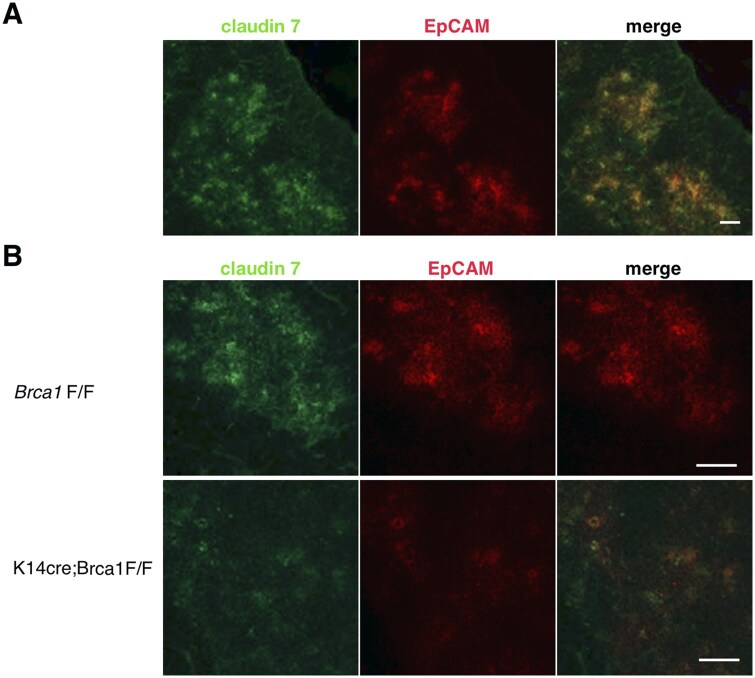
Reduction of EpCAM- and claudin-7–expressing cells in the thymus of BRCA1-deficient mice. (A) Immunofluorescence staining of thymus sections from wild-type C57/6B mice was performed using antibodies against claudin-7 and EpCAM. Claudin-7 extensively colocalizes with EpCAM along mTECs in normal mice. (B) Downregulation of EpCAM/claudin-7 in the mTECs of K14*cre*;*Brca1*F/F mice. Colocalization of EpCAM with claudin-7 is seen exclusively on the mTECs of *Brca1*F/F mice, whereas the expression of EpCAM/claudin-7 is reduced in the mutant mice. Data are representative of independent experiments (*n* = 8 in each group). Scale bars = 100 µm.

Adhesive structures between adjacent cells, including adherens junctions and tight junctions, are not only required for tissue integrity but also act as signaling structures. Tight junctions contain many adhesion molecules, including claudins, occludin, Zonula occludens-1, and junctional adhesion molecules.^32^ Recent studies have shown that EpCAM and claudin-7 directly associate and form a protein–protein complex in various tissues and cultured cells.^33^^,^^34^ When the expression of claudin-7 was examined in thymic tissues of wild-type C57BL/6 mice, claudin-7 was found predominantly in the medulla (Fig. 4A). As expected, the colocalization of claudin-7 and EpCAM was detected. We next asked whether the lack of BRCA1 may affect the expression of EpCAM and claudin-7 in mTECs. EpCAM was highly expressed and extensively colocalized with claudin-7 in mTECs of control mice (Fig. 4B). Notably, EpCAM and claudin-7 were downregulated in BRCA1-deficient mice (Fig. 4B).

### Appearance of β5t-expressing cells in the thymic medulla of BRCA1-deficient mice

**Figure 5.**
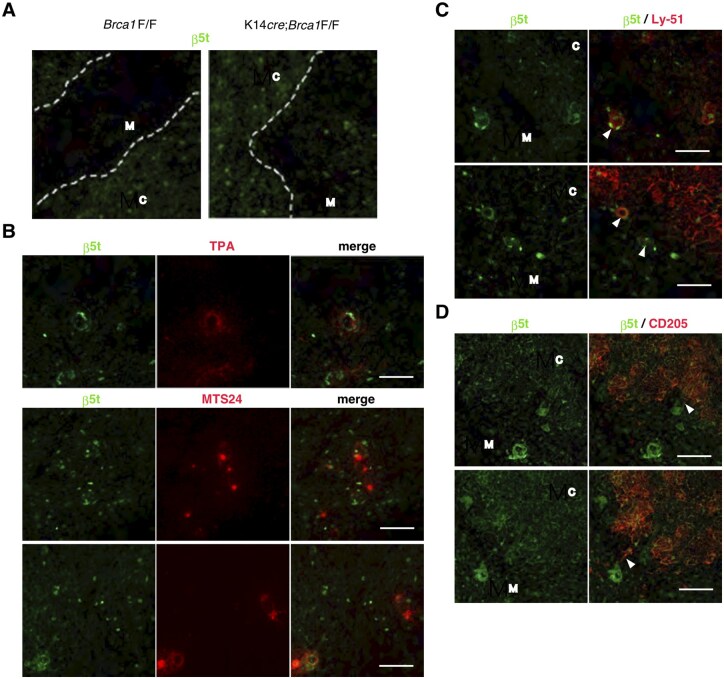
Localization of β5t^+^ cells in thymic medulla of BRCA1-deficient mice. Immunofluorescence staining of thymus sections from *Brca1*F/F and K14*cre*;*Brca1*F/F female mice was performed to detect β5t (A–D) and the binding of TPA or MTS24 (B), Ly-51 (C), or CD205 (D). β5t expression is restricted in cTECs of *Brca1*F/F mice, whereas β5t^+^ cells are also seen in the thymic medulla and often adjacent to Hassall’s corpuscles in BRCA1 knockout mice. Some medullary β5t^+^ cells express Ly-51 (C, arrowheads), and CD205 is expressed in these β5t^+^ cells at the cortical-medullary junction (D, arrowheads). Data are representative of independent experiments (*n* = 8 in each group). C, cortex; M, medulla. Scale bars = 100 µm.

In the mouse thymus, thymoproteasome catalytic subunit β5t (also known as proteasome subunit beta 11) is specifically expressed in approximately 80% of cTECs,^26^ whereas β5t in human is detected not only in cTECs but also in a fraction of thymic dendritic cells.^35^ We examined the expression of β5t in the thymus of K14*cre*;*Brca1*F/F mice and controls. β5t was restricted to the cortex and barely detectable in the medulla of *Brca1*F/F mice (Fig. 5A) and K14*cre* mice (data not shown). Remarkably, an increased number of β5t^+^ TECs was detected in the BRCA1 mutant thymus, and the presence of β5t^+^ cells was also observed in the medulla (Fig. 5A). Interestingly, β5t-expressing cells were frequently localized adjacent to Hassall’s corpuscles in the mutant mice (Fig. 5B–D). In addition, some of them in the medulla occasionally formed small spherical bodies (Fig. 5C, D). Colocalization of β5t and MTS24 was barely detected, indicating that β5t^+^ cells were negative for the putative epithelial progenitor marker MTS24 (Fig. 5B). Moreover, we examined the expression of various markers for cTECs, such as Ly-51 and CD205, in the thymic medulla of the BRCA1 mutant mice. Interestingly, β5t-expressing spherical epithelial structures were often positive for Ly-51 (Fig. 5C). On the other hand, CD205 was expressed in these β5t^+^ cells at the cortical-medullary junction (Fig. 5D).

### BRCA1 deficiency in K14^+^ cells leads to high density of cTECs

**Figure 6.**
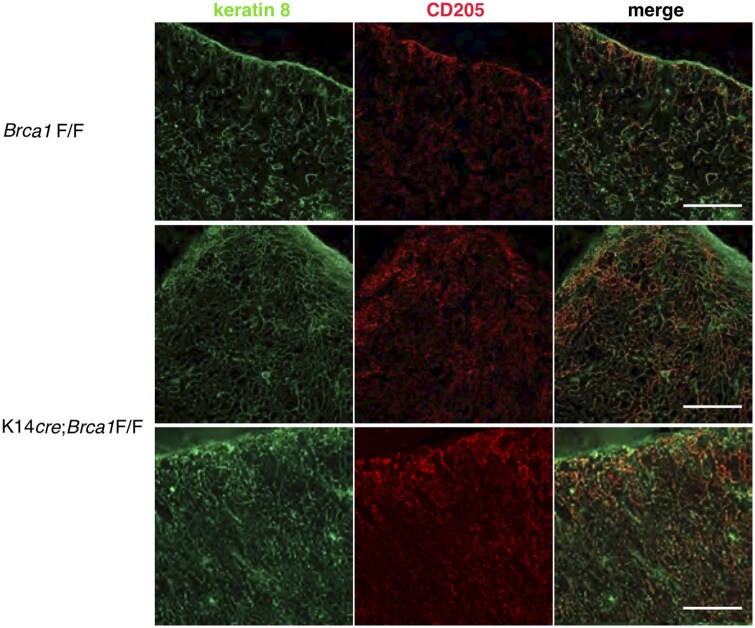
BRCA1 deficiency in K14^+^ cells leads to the expansion of cTECs. Immunofluorescence staining of thymus sections from *Brca1*F/F and K14*cre*;*Brca1*F/F female mice was performed to detect keratin 8 and CD205. Notably, the density of keratin 8^+^CD205^+^ cTECs is higher in K14*cre*;*Brca1*F/F mice than in *Brca1*F/F mice. Data are representative of independent experiments (*n* = 8 in each group). Scale bars = 100 µm.

To further assess the impact of the BRCA1 deficiency on the structure of the cortical epithelium in the thymus, we examined the expression of K8 and CD205 in *Brca1*F/F versus K14*cre*;*Brca1*F/F female mice (Fig. 6). Expression of both markers was restricted to the cortex in both mice. Notably, the density of K8^+^CD205^+^ cTECs was higher in the mutant mice compared to controls, and their thymic cortical epithelium appeared to be cell crowding.

### Thymocyte development in BRCA1-deficient mice

**Figure 7.**
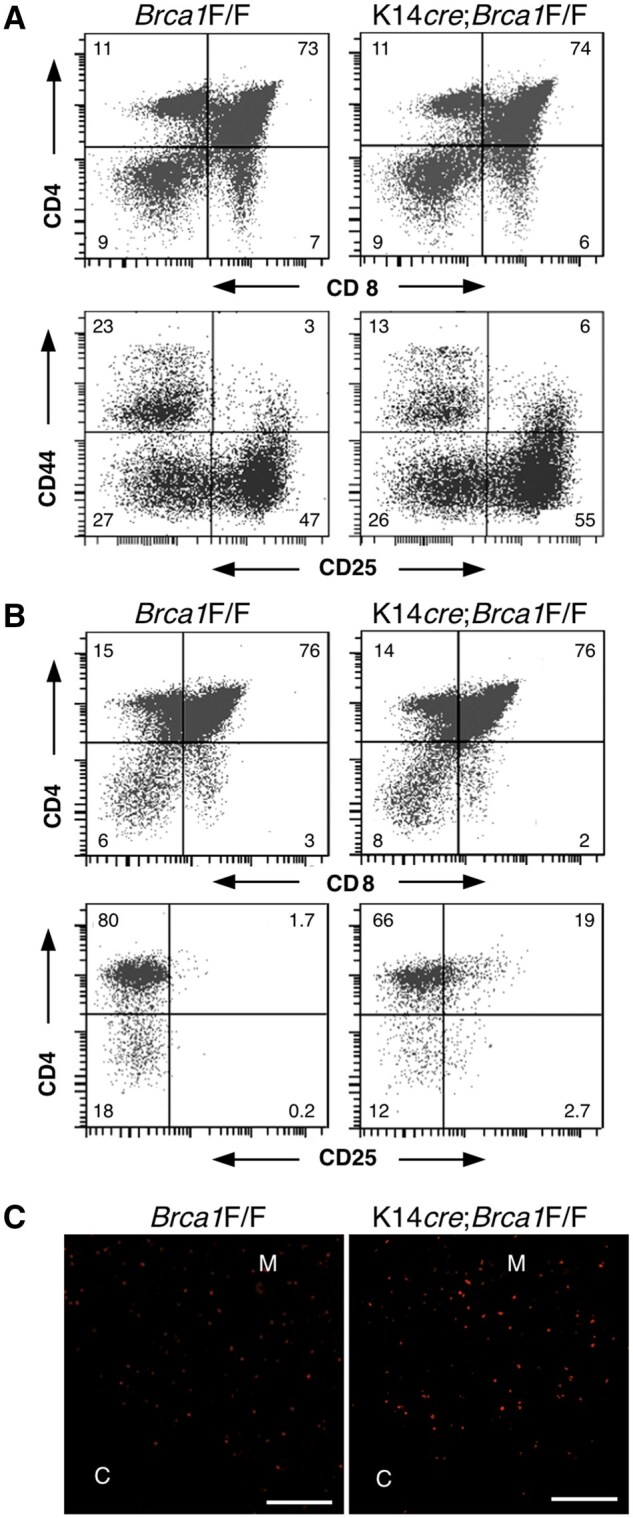
T-cell development in BRCA1-deficient mice. Flow cytometric analyses were performed on thymocytes from *Brca1*F/F *and* K14*cre*;*Brca1*F/F female mice at the age of 5 weeks (A) or 20 weeks (B, C). (A) Thymocytes were stained for CD4 and CD8 (top), and CD3, CD4, and CD8 triple-negative thymocytes from each mouse were stained for CD25 and CD44 (bottom). (B) Thymocytes were stained for CD4 and CD8 (top), and CD3^+^ thymocytes from each mouse were stained for CD4 and CD25 (bottom). The numbers indicate the percentages in each quadrant. The frequency of CD4^+^CD25^+^ thymocytes (mean ± SE, *n* = 4 mice) was 2.1 ± 0.5 and 18.2 ± 2.6 for *Brca1*F/F and K14*cre*;*Brca1*F/F mice, respectively. (C) Immunofluorescence staining of thymus sections from *Brca1*F/F and K14*cre*;*Brca1*F/F female mice was performed to detect Foxp3. Increased generation of regulatory T cells is seen in the thymus of K14*cre*;*Brca1*F/F mice. C, cortex; M, medulla. Scale bars = 100 µm. Data are representative of independent experiments (*n* = 4 in each group).

Previous studies have reported that deletion of BRCA1 in T cells results in a profound depletion of their lineage due to increased thymocyte apoptosis.^36^ To examine the influence of K14-expressing TEC-specific BRCA1 deficiency on T-cell development, thymocytes from *Brca1*F/F and K14*cre*;*Brca1*F/F mice were examined at 5 weeks of age. No differences in thymus size or thymocyte number were observed between controls and BRCA1 mutant mice (data not shown). Flow cytometric analysis of thymocytes revealed the appropriate relative percentages of CD4^+^/CD8^+^ immature thymocytes and single CD4^+^ or CD8^+^ mature thymocytes in both the BRCA1 mutant and control mice (Fig. 7A). In addition, the distribution of the subpopulations of CD4^−^/CD8^−^ thymocytes was similar in mutant and control mice (Fig. 7A).

Hassall’s corpuscles have been shown to instruct dendritic cells to induce the development of CD4^+^CD25^+^ regulatory T cells in human thymus,^37^ suggesting that Hassall’s corpuscles actively communicate with developing T cells in the thymus. We performed flow cytometric analysis for CD4 and CD25 within CD3^+^ thymocytes and found an increase in the proportion of CD4^+^CD25^+^ thymocytes in mutant mice at 20 weeks of age (Fig. 7B). Furthermore, we examined the localization of regulatory T cells in the thymus of *Brca1*F/F versus K14*cre*;*Brca1*F/F mice and found that an increase of Foxp3^+^ thymocytes in K14*cre*;*Brca1*F/F mice was detected at 20 weeks of age (Fig. 7C). The relative frequency of Foxp3^+^ cells (mean ± SE, *n* = 4 mice) was 25.5 ± 3.7 and 76.4 ± 8.8 for *Brca1*F/F and K14*cre*;*Brca1*F/F mice, respectively. Thus, BRCA1 deficiency resulted in increased generation of regulatory T cells.

## Discussion

We found that BRCA1 was expressed in a subset of mTECs that were EpCAM positive, UEA-1 ligand positive, and Aire negative. In previous studies, the mice carrying a T cell–specific disruption of BRCA1 gene show a significant depletion of thymocytes with a developmental defect occurring at a point after the CD4^−^/CD8^−^ stage of thymocyte maturation, suggesting that BRCA1 is expressed in immature thymocytes.^36^ In contrast, we were unable to detect BRCA1 expression in thymocytes of normal C57BL/6 mice by histological approach.

We analyzed thymus tissues from mice in which BRCA1 was deleted specifically in K14-expressing cells. Flow cytometric analysis of thymocytes revealed that thymocyte development appeared phenotypically normal in BRCA1-deficient mice. Interestingly, BRCA1-deficient mice showed enhanced differentiation of mTECs, resulting in the development of Hassall’s corpuscles. We also observed colocalization of EpCAM with claudin-7 in the thymus of control mice. In EpCAM knockout mice, tight junctions in the intestinal epithelium are morphologically abnormal, with the network of tight junction strands scattered and dispersed.^38^ On the other hand, depletion of BRCA1 in K14-expressing cells resulted in downregulation of EpCAM expression along with claudin-7, but no apparent defect for the tight junction network was seen in the thymus of BRCA1 knockout mice (data not shown). Given these results, it is unlikely that BRCA1 mediates the expression of EpCAM and claudin-7 in mTECs. Rather, the frequency of EpCAM^+^claudin-7^+^ mTECs may be reduced due to enhanced development of mTECs into Hassall’s corpuscles in BRCA1-deficient mice.

We found that β5t expression was also sparsely detected in the medullary region of K14*cre*;*Brca1*F/F mice. Notably, β5t^+^ cells were frequently seen adjacent to Hassall’s corpuscles. An association between thymic stromal lymphopoietin-expressing Hassall’s corpuscles and CD11c^+^ dendritic cells has been reported in human.^37^ However, the expression of CD11b and CD11c was not detectable in β5t^+^ cells of BRCA1 mutant mice (data not shown). A fraction of β5t^+^ cells localized in the thymic medulla occasionally expressed Ly-51. CD205 was expressed in some β5t^+^ cells at the cortical-medullary junction, and these β5t^+^ cells appeared to upregulate CD205 expression when they could contact the existing cortical epithelium at the cortical-medullary junction in BRCA1-deficient mice. Furthermore, there was high density of cTECs in the mutant mice. EGFP expression in β5t-Cre × loxP-EGFP mice is detected not only in the cortex but also in the medulla of the thymus.^39^ Based on our results for the morphological structure of medullary β5t^+^ cells, it is likely that medullary β5t^+^ cells appeared to differentiate into cTECs in BRCA1-deficient mice. In addition, cTEC progenitors capable of differentiating into a β5t-expressing cortical lineage may be localized adjacent to Hassall’s corpuscles in BRCA1-deficient mice.

Thus, the deficiency of BRCA1 in K14^+^ TECs led to their impaired development, affecting not only mTECs, but also cTECs. In the mammary gland, there is accumulating evidence for the common progenitor cells in luminal cells and basal/myoepithelial cells, also referred to as bipotent mammary epithelium progenitor cells.^40^ BRCA1 deficiency in mammary epithelium appears to disrupt cell fate specification, with luminal cells being biased toward a more basal-like phenotype. There are 2 possibilities for the appearance of β5t-expressing cTEC progenitor cells in thymus medulla of BRCA1-deficient mice. One possibility is that BRCA1 regulates the cell fate of the progenitors for 2 epithelial subsets in the thymus, similar to the mammary gland. The absence of BRCA1 might bias K14^+^ mTEC lineage toward cTEC lineage, resulting in the expansion of K8^+^ cTECs. The other possibility is that BRCA1 inhibits the differentiation of mTECs into Hassall’s corpuscles. The appearance of β5t-expressing cTEC progenitors may be associated with the development of Hassall’s corpuscles in BRCA1-deficient mice. Although the relationship between 2 TEC progenitors and the development of Hassall’s corpuscles is unknown, we currently favor the latter, as BRCA1 was only expressed in a subset of mTECs. Further experiments are required to determine how BRCA1 expression in mTECs may inhibit their differentiation into Hassall’s corpuscles. In addition, the molecular mechanisms underlying the appearance of cTEC progenitors in BRCA1-deficient thymus will be elucidated. In addition, we provided evidence for an increased generation of thymic regulatory T cells in BRCA1-deficient mice. Our findings have relevance to immune response in patients with BRCA1-linked cancer. Although the immune systems in the carriers with BRCA1 mutation remains to be determined, it is likely that regulatory T cells actively suppress antitumor T-cell responses. The suppressed thymic function will be worse for these patients.

In summary, our present data reveal a key role for BRCA1 in the regulation of TEC differentiation. Although the focus of BRCA1 has historically been on breast and ovarian cancer, BRCA1 has been found to play a novel role in regulating embryonic brain development and postnatal brain size.^41^ Hassall’s corpuscles are not typically seen in mouse but well developed in human.^22^ Nevertheless, it will also be necessary to determine the localization of BRCA1 in human thymus and whether BRCA1 germline mutation might influence thymus structure.

## Data Availability

The data that support the findings of this study are available from the corresponding author upon reasonable request.
